# Ubiquitylation of RUNX3 by RNA-binding ubiquitin ligase MEX3C promotes tumorigenesis in lung adenocarcinoma

**DOI:** 10.1186/s12967-023-04700-8

**Published:** 2024-02-29

**Authors:** Zelai He, Huijun Zhang, Haibo Xiao, Xiangyu Zhang, Hongbo Xu, Ruifen Sun, Siwen Li

**Affiliations:** 1 Department of Radiation Oncology, The first affiliated hospital of Bengbu Medical University, Bengbu, 233000 Anhui China; 2 Department of Cardiothoracic Surgery, Huashan Hospital of Fudan University, Shanghai, 200040 China; 3 Department of Cardiothoracic Surgery, Xinhua Hospital Affiliated to Shanghai Jiao Tong University School of Medicine, Shanghai, 200092 China; 4 Department of Pathology, Jining First People’s Hospital, Jining Medical University, Jining, 272002 Shandong China; 5 Science and Technology Division, Yunnan University of Chinese Medicine, Kunming, 650500 Yunnan China; 6 Department of Thoracic Surgery, The Sixth Affiliated Hospital of Guangzhou Medical University, Qingyuan, 511500 Guangdong China

**Keywords:** LUAD, MEX3C, RUNX3, Suv39H1, Tumor growth, Metastasis

## Abstract

Lung adenocarcinoma (LUAD) is the most common pathological type of lung cancer, but the early diagnosis rate is low. The RNA-binding ubiquitin ligase MEX3C promotes tumorigenesis in several cancers but its mechanism of action in LUAD is unclear. In this study, the biological activity of MEX3C was assessed in LUAD. *MEX3C* and *RUNX3* mRNA levels in the tissues of LUAD patients were determined using reverse transcription‑quantitative PCR. The involvement of MEX3C in the growth and metastasis of LUAD cells was measured by EdU assay, CCK-8, colony formation, Transwell assay, TUNEL, and flow cytometry. Expression of apoptosis and epithelial–mesenchymal transition related proteins were determined using western blotting analysis. LUAD cells transfected with si-MEX3C were administered to mice subcutaneously to monitor tumor progression and metastasis. We found that MEX3C is strongly upregulated in LUAD tissue sections, and involved in proliferation and migration. A549 and H1299 cells had significantly higher levels of MEX3C expression compared to control HBE cells. Knockdown of MEX3C dramatically decreased cell proliferation, migration, and invasion, and accelerated apoptosis. Mechanistically, we demonstrate MEX3C induces ubiquitylation and degradation of tumor suppressor RUNX3. Moreover, RUNX3 transcriptionally represses Suv39H1, as revealed by RNA pull-down and chromatin immunoprecipitation assays. The *in vivo* mice model demonstrated that knockdown of MEX3C reduced LUAD growth and metastasis significantly. Collectively, we reveal a novel MEX3C-RUNX3-Suv39H1 signaling axis driving LUAD pathogenesis. Targeting MEX3C may represent a promising therapeutic strategy against LUAD.

## Introduction

Lung cancer remains the leading cause of cancer-related deaths worldwide with non-small cell lung cancer (NSCLC) attributed to more than 80% of all lung cancer cases [1, 2]. Moreover, NSCLC is seldom detected at an early stage [3]. Most patients with NSCLC are diagnosed at advanced stages and have lymph node and distant organ involvement with high rates of therapy resistance [2, 4]. The diagnosis of NSCLC is further complicated by its heterogeneity into subgroups with different transcription and prognostic profiles. The largest of these subgroups are lung squamous cell carcinoma (LUSC) and adenocarcinoma (LUAD) [5, 6]. Therefore, therapy that focuses on specific targets and mutations to halt the spread of NSCLC may improve survival rates in subsets of patients.

MEX3C (also known as RKHD2) belongs to the MEX3 (Muscle Excess 3) protein family consisting of four members (MEX3A-D) in human [7]. The MEX3 family are important RNA-binding ubiquitin ligases that post-transcriptionally regulate various biological processes [8]. Recent studies demonstrated that MEX-3 proteins are dysregulated in multiple cancers and influence apoptosis, antigen processing and immune evasion of tumor cells, implicating important roles of MEX-3 in tumorigenesis as potential markers and therapeutic targets [9–13]. MEX3C was upregulated in ovarian cancer (OC) tissues, acting as a new oncogene in OC [14]. Moreover, study have demonstrated that MEX3C promotes bladder carcinogenesis via controlling lipid metabolism through the JNK pathway [7]. MEX3A, a homolog of MEX3C, has similarly been demonstrated to have an oncogenic function in LUAD. MEX3A promotes metastasis of lung cancer via interacting with LAMA2 and activating the PI3K/AKT pathway [15]. This raises the question of whether or not MEX3C is likewise carcinogenic in LUAD. In-depth research will be conducted to determine the specific procedure.

The runt-related transcription factors (RUNX) family in mammals consists of three key developmental regulators: RUNX1, RUNX2, and RUNX3, which serve important roles in cell cycle progression, differentiation, apoptosis, immunology, and epithelial–mesenchymal transition (EMT) [16]. RUNX3 has been linked to the development of cancers such as colorectal cancer, liver cancer, lung cancer, and breast cancer [17]. Ubiquitylation is a post-translation modification involved in cell-cycle integrity that has been often implicated in the genome instability that accompanies tumorigenesis including lung cancer [18, 19]. In human gastric cancer, suppressing MEX3B expression inhibits the ubiquitylation and degradation of RUNX3, while the interaction of lncRNA HOTAIR with RUNX3 promotes the MEX3B-dependent ubiquitylation and degradation of RUNX3 [20]. Furthermore, it has been documented that the transcription factor RUNX3 has binding affinity towards the promoter region of SOD3, hence impeding its transcription [21]. Although the ubiquitylation and degradation of RUNX3 has been reported in other cancer types, the role of MEX3C as the specific E3 ubiquitin ligase regulating this process in LUAD remains unknown. We hypothesized that MEX3C induces ubiquitylation of RUNX3, leading to its degradation and driving tumorigenesis in LUAD.

Suppressor of variegation 3–9 homolog 1 (Suv39H1), a SET domain-containing histone methyltransferase, has been reported to participate in tumorigenesis in various types of cancer [22]. Suv39H1 is widely considered as a tumor suppressor due to its activities in inhibiting proliferation-related genes and promoting senescence [23]. However, mounting evidence suggests that Suv39H1 may possibly function as an oncogene in various malignancies, including colon carcinoma, bladder cancer, and hepatocellular carcinoma [24]. Our investigation revealed the existence of binding sites between RUNX3 and Suv39H1. Consequently, we formulated the possibility that the transcription factor RUNX3 may functionally link to Suv39H1 by binding to its promoter region. This potential connection could be important in LUAD pathogenesis and merits further investigation.

In this study, we aim to determine the expression pattern and clinical significance of MEX3C and RUNX3 in LUAD. We hypothesize that MEX3C functions as an oncoprotein that promotes LUAD progression by interacting with tumor suppressor RUNX3, triggering its degradation and transcriptional inactivation. RUNX3 may further regulate downstream targets including Suv39H1 that are involved in LUAD cell activities. Our objectives are to investigate the effects of MEX3C on LUAD cell proliferation, apoptosis, migration and invasion in vitro and tumor growth and metastasis in vivo. We will also elucidate the potential molecular mechanisms focusing on MEX3C-RUNX3 interplay and downstream signaling such as Suv39H1. We expect to establish the role of MEX3C in LUAD and provide evidence that targeting MEX3C could be a promising therapeutic strategy.

## Material and methods

### Clinical samples

From 2018 to 2022, tumor and adjacent tissues were gathered from Huashan Hospital, Fudan University, from 55 LUAD patients, 33 males and 22 females ranging in age from 23 to 76 years. Prior patient consent and approval from the Institutional Research Ethics Committee were obtained (Ethics No.: 2022-490). During the trial, information about patients who did not receive radiotherapy or chemotherapy was documented in their medical records before surgery to ensure that they were followed up properly. Relative gene mRNA expressions in the tumor tissues of LUAD patients were detected by reverse transcription-quantitative polymerase chain reaction (qRT-PCR). Clinical and pathological information was obtained and immunohistochemical studies were performed in paraffin-embedded tissue sections. Table 1 presents a summary of the clinicopathological characteristics of the cases.

**Table 1:** Association between the mRNA Expression of MEX3C with clinical indicators

Clinical data	Patient number (n = 55)	MEX3C Expression		χ^2^ value	P value
		High, n (%)	Low, n (%)		
Sex					
Male	33	16 (48.49)	17 (51.52)	0.7885	0.3746
Female	22	8 (36.37)	14 (63.63)		
Age, year					
≤ 60	21	11 (52.3)	13 (47.7)	0.0836	0.7725
> 60	34	13 (38.2)	18 (61.8)		
Smoking history					
Yes	36	16 (44.4)	20 (55.6)	0.0277	0.8678
No	19	8 (42.1)	11 57.9)		
Histological types					
LUSC	35	15 (42.9)	20 (57.1)	0.0236	0.8744
LUAD	20	9 (45)	11 (55)		
Tumor size, cm					
≤3	23	13 (56.6)	10 (43.4)	2.6686	0.1023
> 3	32	11 (34.4)	21 (65.6)		
TNM stage					
I–II	30	9 (30.0)	21 (70.0)	4.9899	0.0255^*^
III–IV	25	15 (60.0)	10 (40.0)		
LNM					
Yes	23	12 (52.2)	11 (47.8)	1.1715	0.2791
No	32	12 (37.5)	20 (62.5)		
Distant metastasis					
Yes	33	18 (54.5)	15 (45.5)	3.9919	0.0457^*^
No	22	6 (27.3)	16 (72.7)		

55 patients with NSCLC were divided into high-expression (n = 24) and low-expression (n = 31) groups according to the mean value of *MEX3C* mRNA expression

Chi-square test or Fisher’s exact test was performed to evaluate the relationship between MEX3C expression and clinicopathological features of NSCLC

*P < 0.05

### TCGA, GTEx database analysis

RNA-sequencing expression (level 3) profiles and corresponding clinical information for tumors were downloaded from the The Cancer Genome Atlas (TCGA) and Genotype-Tissue Expression (GTEx) dataset [25]. All the analysis methods and R package were implemented by R version 4.0.3. Two-group data performed by wilcox test. P values less than 0.05 were considered statistically significant.

### Cell cultures

Human bronchial epithelial (HBE) cell line BEAS-2B and human LUAD cell lines A549, PC-9, H1975, and NCI-H1299 were obtained from the Cell Bank of the Chinese Academy of Sciences (Beijing, China). HBE cells were grown in DMEM culture media (Invitrogen, Gaithersburg, CA, USA). A549, H1650, H838 and H1299 cell lines were grown in RPMI-1640 medium (Invitrogen) supplemented with 10% fetal bovine serum (FBS, Invitrogen) and 1% penicillin/streptomycin (P/S, Sigma-Aldrich, Steinheim, Germany) solution, in an incubator with 5% CO_2_ at 37 ℃. HEK293 cells were purchased from the Chinese Academy of Science Cell Bank (Shanghai, China). HEK293 cells were grown in DMEM (Invitrogen) with 10% FBS, 100 U/ml penicillin, and 100 μg/ml streptomycin at 37 °C in a humidified atmosphere of 5% CO_2_.

### Cell transfection

siRNAs specific for MEX3C, E-cadherin, and RUNX3 as well as the corresponding scrambled siRNA were purchased from GenePharma (Shanghai, China). MEX3C and RUNX3 overexpression plasmid (OE-MEX3C and OE-RUNX3) and the NC plasmid (an empty vector) were generated using the pcDNA3.1 vector purchased from GenePharma company (Shanghai, China). The A549 and H1299 cells were seeded on coverslips in 6-well dishes at a density of 3 × 10^6^ cells per well, and they were allowed to attach in a culture medium for a period of 24 h. Overexpressing plasmid (2 μg) or siRNA (1.5 μg) of indicated genes were transfected into cells using Lipofectamine 3000 (Invitrogen) according to the manufacturer’s instructions, which were described for over-expression and knockdown of indicated genes. The selected sequences for knockdown as follow: si-MEX3C-1: 5′-GGCUAAAGUUGUUAGUAAACU-3′, si-MEX3C-2: 5′-AGUUGUUAGUAAACUUAUAAA-3′; si-MEX3C-3: 5′-GGUCAGUAUUGAAACCUAAUC-3′; si-RUNX3-1: 5′- GAUUUGUUACAAUAAUAUAAC-3′, si-RUNX3-2: 5′-GCUCUGUGAUUAUAAGCAACA-3′, si-RUNX3-3: 5′-GACUGAUUUGUUACAAUAAUA-3′; si-E-cadherin-1: 5′- GAGUAAGUGUGUUCAUUAAUG-3′, si-E-cadherin-2: 5′- GUGUGUUCAUUAAUGUUUAUU-3′, si-E-cadherin-3: 5′- GGAGUUCUCUGAUGCAGAAAU-3′. The expression efficiency was detected by qRT-PCR and western blotting analysis after transfection.

### Cell counting kit-8 (CCK-8) assay

LUAD cell viability was determined by CCK-8 assays. After transfection, cells (4 × 10^3^ cells/well) that had been transfected were seeded in DMEM (Capricorn Scientific, USA) supplemented with 10% fetal bovine serum (FBS, Capricorn Scientific, USA) for 24, 48, 72, 96, or 120 h. The cell suspension was then allowed to remain in an environment containing 20 μL of CCK-8 for a period of 4 h. After then, 150 μL of DMSO was added to the medium. After 10 min, cell viability was assessed using a microplate reader (Olympus Corporation, Tokyo, Japan) to determine the optical density at 490 nm.

### Flow cytometry

The annexin V-fluorescein isothiocyanate (FITC)/propidium iodide (PI) apoptosis detection kit (Beijing Biosea Biotechnology, China) was used in conjunction with flow cytometry analysis to determine the percentage of cells that had undergone the process of apoptosis. Fixed cells were then washed twice in PBS and stained in PI/FITC-annexin V in the presence of 50 μg/ml RNase A (Sigma-Aldrich). The apoptotic cell rate was measured using a FACS can after an incubation period of 2 h at room temperature in the absence of light (Beckman Coulter, USA). FlowJo was utilized in order to perform the statistical analysis on the data (TreeStar, USA).

### Terminal deoxynucleotidyl transferase dUTP nick-end labeling (TUNEL) assay

TUNEL assay (Roche, Indianapolis, IN, USA) was used to measure apoptosis following the manufacturer’s instructions. Briefly, 3 × 10^5^ cells were seeded onto a cover slip in a 6-well plate and attached overnight. Then, cells were treated as indicated followed by 4% formaldehyde-PBS fixation for 15 min at room temperature, after which they were permeabilized with 0.2% Triton X-100 in PBS for another 10 min under the same conditions. After being washed twice with PBS, the cells were incubated with a fluorometric terminal deoxytransferase mixture at 37 °C for 1 h. After three washes with PBS, cover slips were mounted with Vectashield Antifade Mounting Medium (Vector Laboratories) containing DAPI, to counterstain cellular nuclei. Fluorescence images were captured in at least five views using a Nikon Eclipse Ti-E fluorescence microscope (Nikon Corporation, Tokyo, Japan).

### EdU (5-ethynyl-2′-deoxyuridine) incorporation assay

Cell proliferation was measured by EdU assay using the Cell-LightTM EdU Apollo^®^567 In Vitro Kit (RiboBio, Guangzhou, China) according to the manufacturer’s protocol. Briefly, 1 × 10^5^ cells were incubated with 10 μM EdU for 2 h before fixation with 4% paraformaldehyde, permeabilization with 0.5% Triton X-100 and EdU staining. Cell nuclei were stained with DAPI at a concentration for 10 min. The number of EdU-positive cells was counted under a fluorescence microscope in five random fields (Olympus IX53; Olympus, Tokyo, Japan). ImageJ software was used to quantify fluorescence levels.

### Transwell assay

After the cells were transfected, 5 × 10^4^ of A549 and H1299 cells in serum-free medium were plated on uncoated upper chambers (Merck Millipore) for migration tests and Matrigel-coated upper chambers (BD Bioscience, USA) for invasion assays, respectively. These steps were repeated for invasion assays. After an additional twenty-four hours had passed, the lower wells received the culture medium that was 10% FBS containing. After that, a cotton swab was used to remove the cells that had not invaded or migrated from their original location. After that, the filters were first steeped in ethanol (90%) for 10 min, and then crystal violet was used to dye them for the following 15 min. The utilization of a microscope with its objective turned upside down made it possible to count five random fields in each chamber (Leica, Germany). Each experiment was repeated three times.

### Colony formation assay

For the colony formation assay, cells were seeded at a density of 1 × 10^3^ cells/well. Cells were treated with 150 μM CoCl_2_, and seeded in each well of a 6-well cell culture plate. After 2 weeks, they were fixed in 4% paraformaldehyde and stained with 1% crystal violet. The colony numbers were counted to assess cell proliferation. The assays were performed in three independent experiments.

### Hematoxylin and eosin (H&E) staining

Tumor or lung tissues were fixed in 4% paraformaldehyde overnight and then embedded in 4% paraffin overnight at 4 °C. 4 μm thick tissues sections were stained by using H&E for histological analysis under a light microscope.

### Immunohistochemical staining

For immunohistochemistry analysis, tumor tissue sections (5 µm) were dewaxed with a gradient alcohol series and incubated with goat serum for 30 min at 37 °C. The sections were stained for primary antibody overnight at 4 °C. The primaery antibodies used were as follows: MEX3C (Abcam, Ab243457, 1:150), RUNX3 (Cell signaling, #9647, 1:100), E-cadherin (Abcam, ab231303, 1:50), N-cadherin (Abcam, ab207608, 1:50), anti-Ki-67 (Abcam, ab15580, 1:100), and anti-PCNA (Abcam, ab29, 1:50). Thereafter, sections were incubated with secondary anti-IgG antibody and incubated at 37 °C for 30 min. Finally, sections were stained by a DAB (3,3′-diaminobenzidine) substrate kit (Dako, Carpinteria, CA, USA), counterstained with hematoxylin, and observed microscopically under a microscope.

### Wound healing assays

To assess the migration of cells, LUAD cells were seeded onto six-well plates (3 × 10^6^ cells/well) and incubated at 37 °C in 5% CO_2_ for 48 h in RPMI-1640 medium containing 2% FBS. The surface of the cells was scratched with a 200 μL tip and then washed twice with PBS to remove detached cells. The cells were then cultured for indicated hours in RMPI-1640 supplemented with 2% FBS, to minimize cell proliferation during the period of assay. The image of each scratch at the same location was captured after the indicated incubation time using an optical microscope (IX53, Olympus, Tokyo, Japan) and assessed using ImageJ software.

### Treatment with proteasome inhibitor

1 × 10^6^ LUAD cells were transfected by Lipofectamine 2000. After overnight culture, the culture medium was replaced with fresh medium containing proteasome inhibitor MG132 (10 μm, MedChemExpress, Shanghai, China). The cells were further cultured at 37 °C with the proteasome inhibitor for 24 h. The cells were then harvested for western blot analysis.

### qRT‐PCR analysis

Total RNA was extracted from cells with TRIzol reagent (TransGen Biotech, Beijing, China). RNA was reverse transcribed using a TransScript All-in-One First-Strand cDNA Synthesis Kit (TransGen Biotech). The resulting cDNA was amplified using a reaction mix containing 10 μL of SYBR Green qPCR Master Mix (TransGen Biotech). The PCR conditions were a denaturation step at 94 °C for 5 min, followed by 40 cycles of 94 °C for 30 s, 58 °C for 30 s, and 72 °C for 30 s. The primer sequences can be found in Table 2. The GAPDH gene was used as an internal control and the relative level of expression was determined using the 2^−ΔΔct^ method.

**Table 2:** Primers used in the RT-qPCR

Gene	Forward	Reverse
*MEX3C*	GAAAGAGCGTCAACACCACC	AAATGGGCTCTTCACCACGA
*RUNX3*	AGCACCACAAGCCACTTCAG	GGGAAGGAGCGGTCAAACTG
*Suv39H1*	CCTGCAGGTGTACAACGTCT	ATCAAAGGTGAGCTCCTCGC
*CEA*	TTACCTTTCGGGAGCGAACC	GTGTGTGTTGCTGCGGTATC
*SCCAg*	GATGCAGACCTCTCAGGCAT	AATCCTACTACAGCGGTGGC
*Ki-67*	GGAAGCTGGACGCAGAAGAT	CAGCACCATTTGCCAGTTCC
*PCNA*	CCTGAAGCCGAAACCAGCTA	TGAGTGCCTCCAACACCTTC
*GAPDH*	CCAGCAAGAGCACAAGAGGA	ACATGGCAACTGTGAGGAGG

### Ubiquitination assay

For in vivo ubiquitination assay, HEK-293 cells were co-transfected with Flag-RUNX3 (1 μg), HA-ubiquitin (2 μg), and Myc-MEX3C (1 μg) expression plasmid using Lipofectamine 3000 (Invitrogen). Immunoprecipitates with anti-Flag agarose were analyzed via immunoblotting with anti-Flag and Myc antibodies. 48 h after transfection, cells were harvested and split into two aliquots, one for immunoblotting and the other for ubiquitination assay. For ubiquitination analysis, A549 and H1299 cells were transfected with His-Ub and MEX3C overexpression plasmid, immunoprecipitation and immunoblot analysis were performed using anti-RUNX3 and anti-ubiquitin antibody (1:1,000; Sigma-Aldrich, China), respectively.

### Luciferase reporter assay

A549 and H1299 cells were co-transfected with RUNX3-OE plasmid or empty vector control and Suv39H1-WT or -MUT. Lipofectamine 2000 (Invitrogen) was used for transfection. After 48 h of transfection at 37 °C with the luciferase reporter vector (Promega), a Dual Luciferase Reporter Assay kit (Promega) was used to evaluate the relative luciferase activities. The Renilla luciferase reporter was used as internal control. The activities of firefly luciferase and Renilla luciferase were quantified by using the dual luciferase reporter assay system (Promega).

### Co-immunoprecipitation (IP) assay

For Co-IP assay, A549 and H1299 cells with or without transfection were collected through an ice‐cold PBS wash and plated on 10 cm dishes and lysed in a lysis buffer containing 1% Triton X-100, 150 mM NaCl_2_, 1.5 mM MgCl_2_, 50 mM HEPES pH 7.6, 1 mM EDTA, 10% glycerol, 10 mM NaF, 1 mM NaVO_3_, 10 mM β-glycerolphosphate, 50 ml DDM, and 5 protease inhibitor tablets and further centrifuged at 12,000 ×*g* at 4 °C for 10 min. Cell lysates were incubated with protein A and/or protein G agarose beads (Santa Cruz Biotechnology) conjugated with specific antibodies for target protein and incubated overnight at 4 °C. Proteins beads were washed three times and boiled with 2 × SDS sample buffer for 10 min at 95 °C, then analyzed by western blotting to detect levels of MEX3C and RUNX3.

### Chromatin immunoprecipitation IP (ChIP) assay

ChIP assays were done on A549 and H1299 cells using a SimpleChIP^®^ Enzymatic Chromatin IP Kit in accordance with the protocol provided by the manufacturer. Following the collection of crosslinked chromatin DNA and its subsequent sonication into fragments ranging from 200 to 1000 bp, the sample was immunoprecipitated with either an RUNX3 antibody (#18,113, Cell Signaling Technology) or a control IgG antibody (#3900, Cell Signaling Technology). After the addition of magnetic beads, the pieces of precipitated chromatin were cleaned, separated, and quantified by using qRT-PCR.

### RNA pull-down assays

LUAD cells (1 × 10^6^) seeded in 6-well plate were transfected with biotinylated RUNX3 and NC (biotin-NC) using Lipofectamine 3000 (Invitrogen). The transfected cells were collected after 48 h following a 10-min lysis buffer treatment at room temperature (25 °C). The cell lysates were then incubated with M-280 Streptavidin magnetic beads (Invitrogen) for 3 h at 4 °C to pull down the biotinylated RNAs and associated proteins. Afterwards, the beads were washed with ice-cold lysis buffer to remove unbound components. The level of Suv39H1 protein enriched by RUNX3 pull-down was measured by qRT-PCR.

### Immunofluorescence assay

The A549 and H1299 cells in a 6-well plate at a density of 3 × 10^5^ cells/well and rested overnight. The slides were then permeabilized by 1% Triton X-100 in PBS for 10 min and blocked in 10% normal goat serum, followed by the incubation with the Cleaved-caspase-3 (Cell signaling, #9661, 1: 400) primary antibody overnight. They were then rinsed 3 times with PBS, and then the secondary antibody was incubated on the coverslips at room temperature for 1 h. The sections were stained by DAPI and observed under a fluorescence microscope.

### In vivo xenograft metastasis assay

SCID mice (6–7 weeks old, male) were purchased from Shanghai Experimental Animal Center of Chinese Academic of Sciences (Shanghai, China), housed in specific pathogen-free conditions with 12 h day/12 h night. The use of animals was approved by the Institutional Animal Care and Use Committee at Huashan Hospital of Fudan University, and all studies were conducted in compliance with the Committee's Guidelines for Animal Care. MEX3C knockdown or scramble control (si-NC) transfected A549 and H1299 cells were injected subcutaneously (3 × 10^6^ cells/100 μL PBS per mouse) into the left flanks of mice. Tumor size was recorded every week using a caliper. Tumor volumes were calculated based on the following formula: Volume (mm^3^) = (L × W^2^)/2, with L being the largest diameter (mm) and W being the smallest diameter (mm). After 6 weeks, the mice were humanely sacrificed. Images were obtained using an Animal Vivo Imaging Machine (Perkin Elmer, Waltham, MA, USA). The tumors were collected for weighing, H&E staining, western blotting and IHC analysis.

In order to create a model of experimental metastasis, mice were split into four groups (n = 5 per group), and then a mixture of resuspended A549 and H1299 cells (2 × 10^6^ cells per 100 μL PBS) transfected with either si-NC or si-MEX3C and tagged with luciferase was injected into the tail vein. Bioluminescent imaging using the IVIS image system was used to check for lung metastatic progression weekly. After 8 weeks mice were killed and the lungs were removed and embedded in paraffin for stained with H&E or TUNEL staining. Under a microscope, the number of lung nodules caused by metastasis was tallied.

### TUNEL assay in tumor tissue

The tissue that had been removed from the tumor was deposited in a solution containing 4% paraformaldehyde for the purposes of fixing, routine sectioning, and finally deparaffinizing to water. In order to retrieve the antigen, a working solution of proteinase K was added to the samples, and then they were left to fix at room temperature for 15–30 min. After that, 50 µL of 3% H_2_O_2_ was added, and the mixture was left to incubate at room temperature for 10 min. After that, 50 µL of TUNEL reaction solution was added, and the mixture was left to incubate at 37 °C in the dark for 60 min. Finally, the sample was rinsed three times with PBS for 5 min each time. After that, 50 µL of peroxidase buffer solution was added, and the mixture was allowed to react at 37 °C for 30 min. After that, the sample was rinsed three times with PBS, developed with DAB, and stained in accordance with the instructions that came with the TUNEL kit (Invitrogen).

### Western blot analysis

Cells and tissue were first lysed with RIPA buffer (Beyotime Biotechnology, Shanghai, China) at room temperature for 1 h. The bicinchoninic acid method was used to measure the level of proteins. Proteins in the lysate were separated by electrophoresis and then transferred to a polyvinylidene difluoride membrane. The membrane was blocked with 5% skimmed milk powder (Sigma-Aldrich) and then incubated with primary antibodies overnight at 4 °C. The following antibodies were used: anti-MEX3C (Cell signaling, #50,844, 1:1,000), anti-RUNX3 (Cell signaling, #9647, 1:1,500), anti-Suv39H1 (Novus, NBP1-21,367, 1:1,000), anti-E-cadherin (Abcam, ab231303, 1:1,500), anti-N-cadherin (Abcam, ab76011, 1:1,000), anti-Bcl-2 (Abcam, ab182858, 1:1,000), anti-Bax (Abcam, ab32503, 1:1,000), anti-Cleaved-caspase-3 (Cell signaling, #9661, 1:1,000), and anti-GAPDH (Abcam, ab8254, 1:2,000). The membranes were then washed in TBST and incubated with horseradish peroxidase-labeled secondary antibody. After washing in TBST again, protein bands were visualized using an ECL detection agent (GE Healthcare, Chicago, IL, USA) and ImageJ software. GAPDH were used as internal loading controls.

### Statistical analysis

Three individual replicates were performed for each experiment, and the data were presented by mean ± SD. The statistical analysis was conducted by SPSS 22.0 (SPSS Inc., IL, USA) and Prism 9.0 (GraphPad Software, La Jolla, CA, USA). Paired data were analyzed using the paired Student's t-test. One-way ANOVA followed by Dunnett’s multiple comparison test was used to evaluate significant differences between multiple groups. Chi-square test was performed to evaluate the relationship between MEX3C expression and clinicopathological features. The prognostic value was calculated by the Kaplan–Meier analysis with log-rank test. P < 0.05 was considered statistically significant.

## Results

### Upregulation of MEX3C and downregulation of RUNX3 in LUAD

**Fig. 1:**
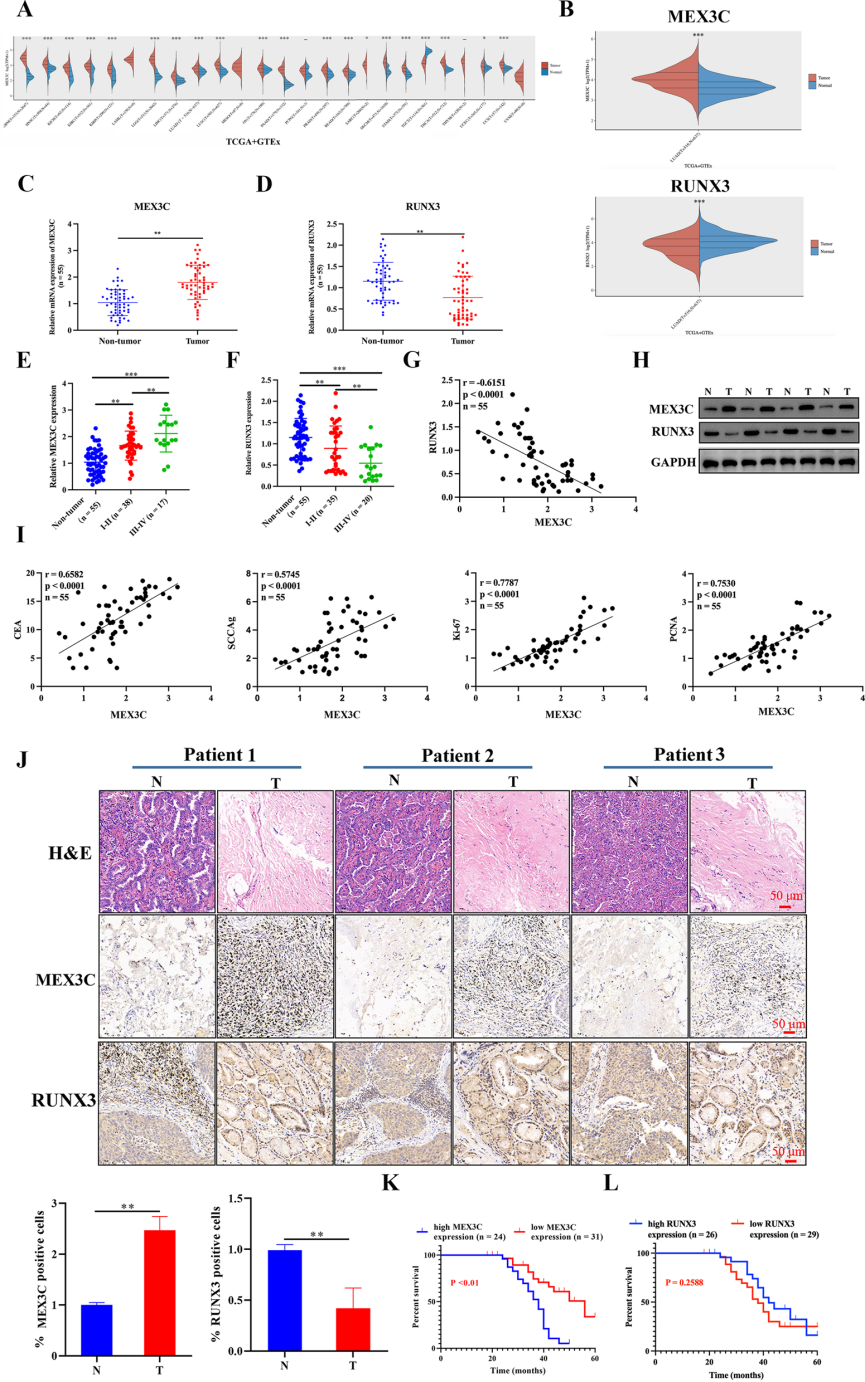
Upregulation of MEX3C and downregulation of RUNX3 in LUAD. **A** The expression distribution of MEX3C gene in 24 type of tumor tissues and normal tissues. The abscissa represents different tumor tissues, and the ordinate represents the expression distribution of gene. **B** *MEX3C* and *RUNX3* mRNA expression of tumor and normal tissues in LUAD datasets were analyzed using the TCGA + GTEx databases. **C**, **D** qRT-PCR was used to detected the MEX3C and RUNX3 mRNA levels in LUAD tissues and paired non-tumor tissues, n = 55. **E**, **F** MEX3C and RUNX3 mRNA expression in LUAD tissues at different stages and healthy subjects were detected using qRT-PCR. **G** Relative *MEX3C* mRNA levels were negatively correlated with relative *RUNX3* mRNA levels in the LUAD tissues (r = − 0.6151, p < 0.0001). **H** Protein levels of MEX3C and RUNX3 in 3 pairs of clinical lung cancer specimens detected by western blotting. T and N indicate lung cancer tissue and paired adjacent normal tissue, respectively, n = 4. **I** Correlation analysis of *MEX3C* with *CEA*, *SCCAg*, *Ki-67*, and *PCNA* mRNA levels in the LUAD tissues via Pearson analysis. **J** H&E and IHC staining of MEX3C and RUNX3 in 3 pairs of NSCLC and adjacent samples, n = 3. Quantification of IHC staining. **K**, **L** Prognostic analysis of MEX3C and RUNX3 for LUAD. Bars represent mean ± SD. *P < 0.05; **P < 0.01

We evaluated the expression distribution of MEX3C between tumor and normal tissues in TCGA + GTEx pan-cancers dataset. The pan-cancer profiles analysis showed that MEX3C expressed at relatively higher levels in most kinds of tumor tissues than in normal tissues (Fig. 1A). Particular, the expression level of MEX3C is significantly higher in LUAD tissues than normal tissues. In contrast, RUNX3 was downregulated in LUAD tumor tissues (Fig. 1B). We next examined *MEX3C* and *RUNX3* mRNA level in 55 paired LUAD tissues and normal paraneoplastic tissues. As shown in Fig. 1C, D, *MEX3C* mRNA levels were higher in LUAD cancer tissues than in normal paraneoplastic tissues, while RUNX3 expression trended in the opposite direction. Besides, the mRNA expression of *MEX3C* increased with tumor grade was the highest in stages III to IV (Fig. 1E). Inversely, the mRNA expression of *RUNX3* in LUAD tissues decreased with tumor grade (Fig. 1F) and a significantly negative correlation was obtained by Pearson analysis between the mRNA expression of *MEX3C* and *RUNX3* (*P* < 0.0001, *r* = − 0.6151) (Fig. 1G). The results of western blotting on protein levels also reveal that MEX3C is elevated in LUAD, while RUNX3 is downregulated (Fig. 1H). Carcinoembryonic antigen (CEA) represents the earliest marker for the diagnosis of NSCLC and squamous cell carcinoma antigen (SCCAg) is associated with the stage of NSCLC [26]. It was discovered that the mRNA expression of *MEX3C* in LUAD tissues correlated with *CEA* and *SCCAg* as well as proliferation markers (*Ki-67* and *PCNA*) [27], indicating its involvement in the growth of LUAD (Fig. 1I). Patients were divided into low expression (n = 31) and high expression (n = 24) groups based on the mean values of *MEX3C* mRNA expression in LUAD tissues. The relationship between MEX3C expression and pathological characteristics was studied. High MEX3C expression was correlated with TNM stage (*P* = 0.0255) and distant metastases (*P* = 0.0457) (Table 1). H&E staining and immunohistochemical analysis of MEX3C and RUNX3 in 3 pairs of LUAD tissues indicate consistently that MEX3C is overexpressed and RUNX3 is under-expressed in LUAD (Fig. 1J). A prognostic analysis of MEX3C was performed on the 55 patients who were followed up on a consistent basis every 2 months. In total, 32 patients passed away during the course of the study, with 24 of those deaths occurring in the groups with high MEX3C expression. A decreased rate of survival was seen in LUAD patients with high expressions of MEX3C (Fig. 1K). Afterward, the expression of RUNX3 and its relationship with prognosis was examined. According to the mean level of *RUNX3* mRNA, patients were divided into high (n = 26) and low (n = 29) groups. Results showed no correlation between RUNX3 expression and prognosis. This further validates the role of MEX3C as a prognostic biomarker for LUAD.

### Knockdown of MEX3C in LUAD cells suppresses migration and invasion in vitro

**Fig. 2:**
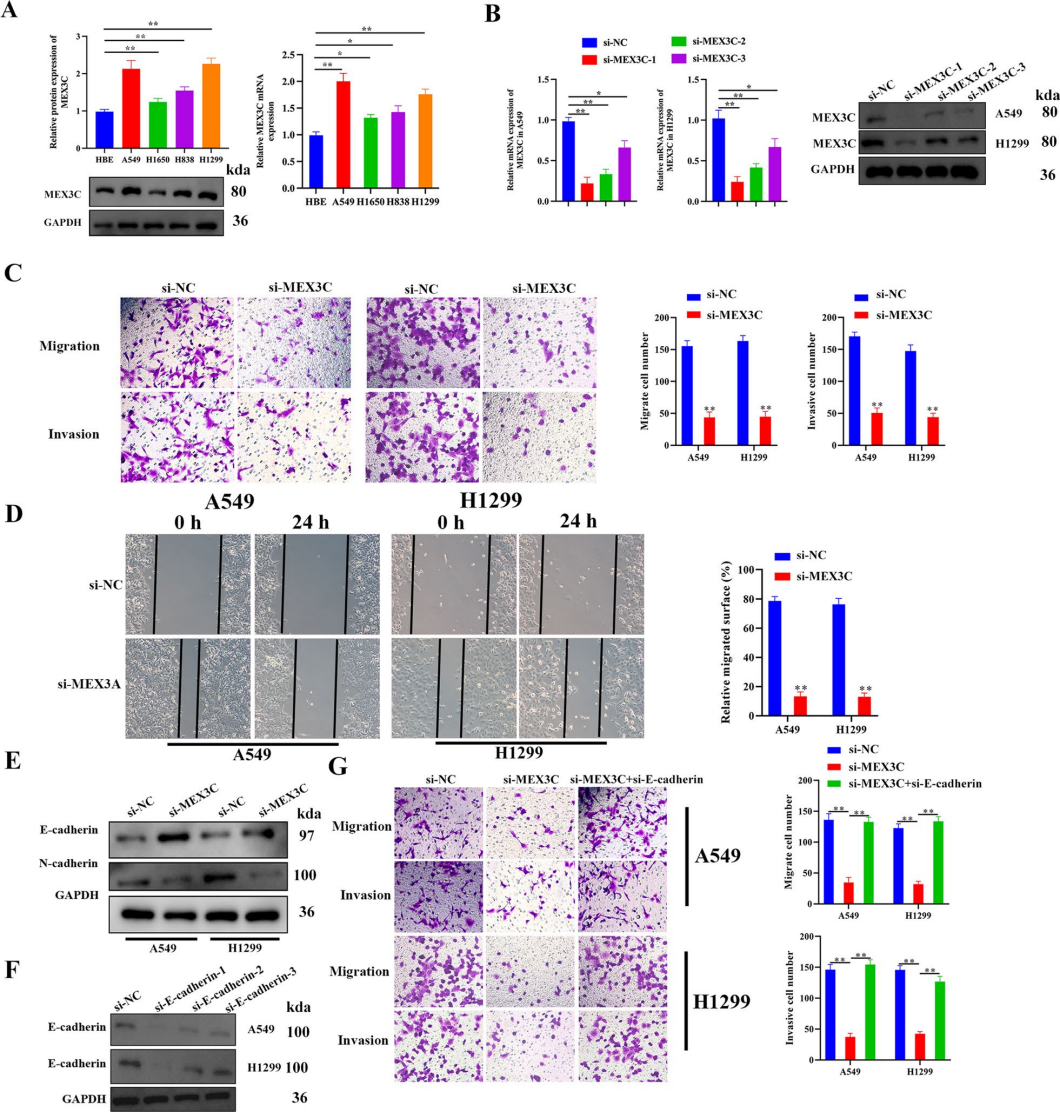
Knockdown of MEX3C in LUAD cells suppresses migration and invasion in vitro. **A** MEX3C mRNA and protein expression in human LUAD cell lines (A549, H1650, H838, and H1299) and a normal human bronchial epithelial cell-line HBE were analyzed by qRT-PCR and Western blotting, respectively. **B** The transcription of MEX3C in A549 and H1299 cells transfected with si-NC lentivirus or si-MEX3C lentivirus for 48 h was detected by qRT-PCR and western blotting analysis. **C** Cell migration and invasion of A549 and H1299 cells treated with or without knockdown of MEX3C were evaluated by Transwell assay. **D** A wound healing assay was used to assess the cell migration ability of A549 and H1299 cells treated with si-NC or si-MEX3C for 24 h. **E** EMT-related proteins E-cadherin and N-cadherin expression were detected by western blotting analysis. **F** Knockdown E-cadherin in A549 and H1299 cells using siRNA, E-cadherin expression was detected by western blotting. **G** The knockdown of E-cadherin was able to reverse the effect of MEX3C suppression on the migration and invasion of LUAD cell lines. Bars represent mean ± SD from 3 independent experiments. *P < 0.05, **P < 0.01

An assessment of the levels of *MEX3C* mRNA and protein in human LUAD cell lines (A549, H1650, H838, and H1299) and a normal HBE cells by qRT-PCR and western blotting analysis, provide a conclusion of MEX3C expression differed between LUAD cell lines, with highest expression in A549 and H1299 (Fig. 2A). To further analyze the relationship between tumor progression and MEX3C, its transcription was silenced in A549 and H1299 cells and verified with qRT-PCR and western blotting analysis, si-MEX3C-3 showed the highest interference efficiency in both A549 and H1299 cell lines and was picked up for further experiments and named si-MEX3C (Fig. 2B). Wound healing and Transwell assays indicated that the knockdown of MEX3C suppresses levels of migration and invasion in the LUAD cell lines (Fig. 2C, D). EMT is a characteristic of tumor progression and is indicated by the upregulation of N-cadherin and consequential downregulation of E-cadherin [28]. The suppression of MEX3C in LUAD cells reduced the levels of EMT as indicated by a higher level of E-cadherin and a lower level of N-cadherin (Fig. 2E). However, the knockdown of E-cadherin by siRNA was able to reverse the effect of MEX3C suppression on the migration and invasion of the A549 and H1299 cell lines (Fig. 2G).

### Knockdown of MEX3C in LUAD cells suppresses proliferation while promotes apoptosis

**Fig. 3:**
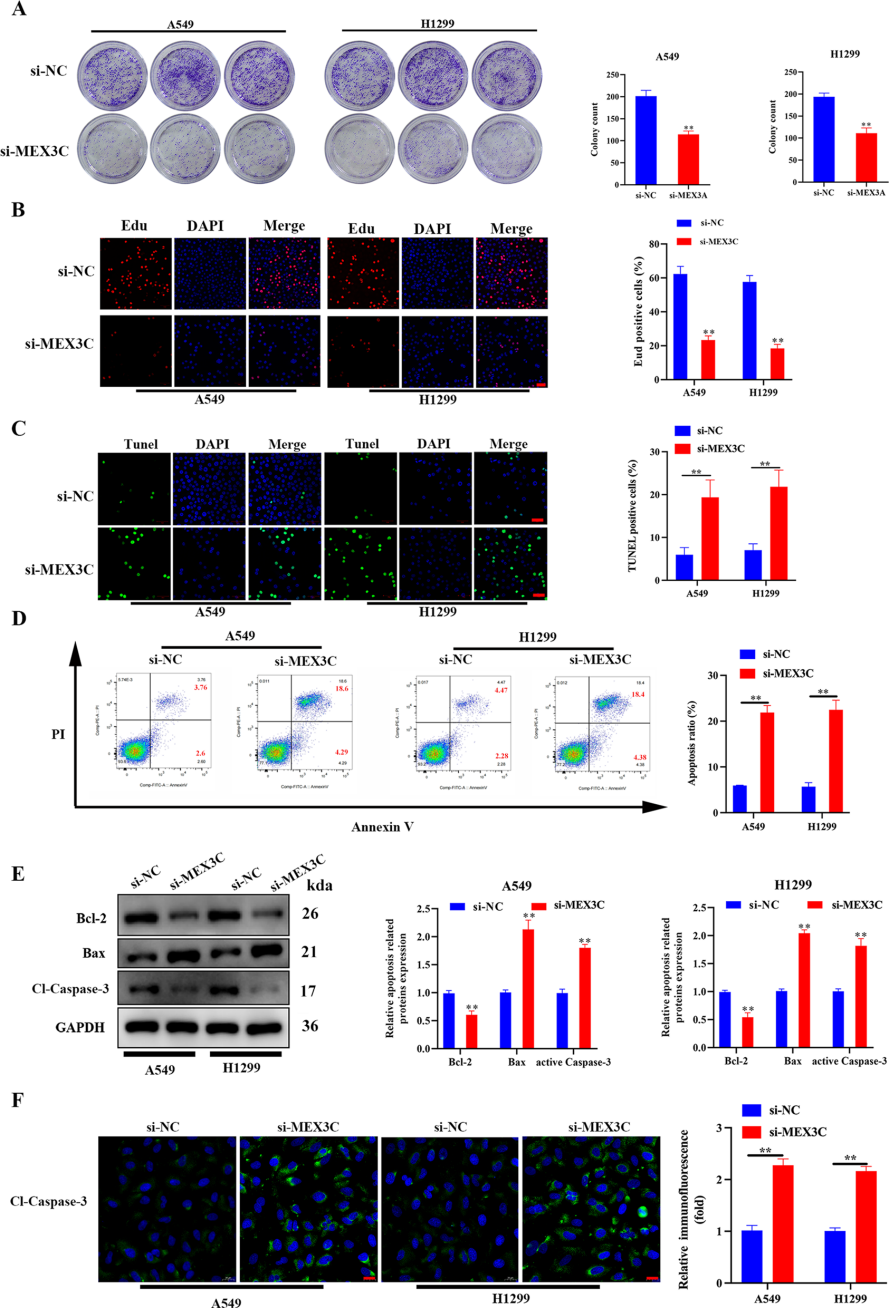
Knockdown of MEX3C promotes apoptosis and inhibits proliferation in LUAD cells. **A** Colony formation was used to evaluated proliferation for A549 and H1299 cells with or without MEX3C knockdown. **B** Proliferation of A549 and H1299 cells was detected using an Edu assay, scale bar, 50 μm. **C** TUNEL staining was used to determine the potential impact of MEX3C knockdown on apoptosis in A549 and H1299 cells, scale bar, 50 μm. **D** Flow cytometry analysis was performed to measure the percentage of apoptotic cells in A549 and H1299 cells after indicated treatment. **E** The expression levels of apoptosis-related proteins (Bcl-2, Bax, and Cleaved-Caspase-3) in MEX3C-silencing A549 and H1299 cells were detected by western blot. **F** Expression of Cleaved-Caspase-3 in A549/H1299 cells as analyzed by immunofluorescence assay, scale bar, 20 μm. Bars represent mean ± SD from 3 independent experiments. **P < 0.01

Further evaluation of MEX3C suppression in A549 and H1299 cells by colony formation, EdU staining, TUNEL staining, flow cytometry, and the expression of apoptosis-related proteins confirmed a reduction in proliferation and increased apoptosis (Fig. 3A–E). Altogether, these findings indicate that Knockdown of MEX3C promotes apoptosis and inhibits proliferation in LUAD cells, MEX3C plays a substantial role in the tumor progression of LUAD.

### MEX3C mediated RUNX3 ubiquitination and degradation

**Fig. 4:**
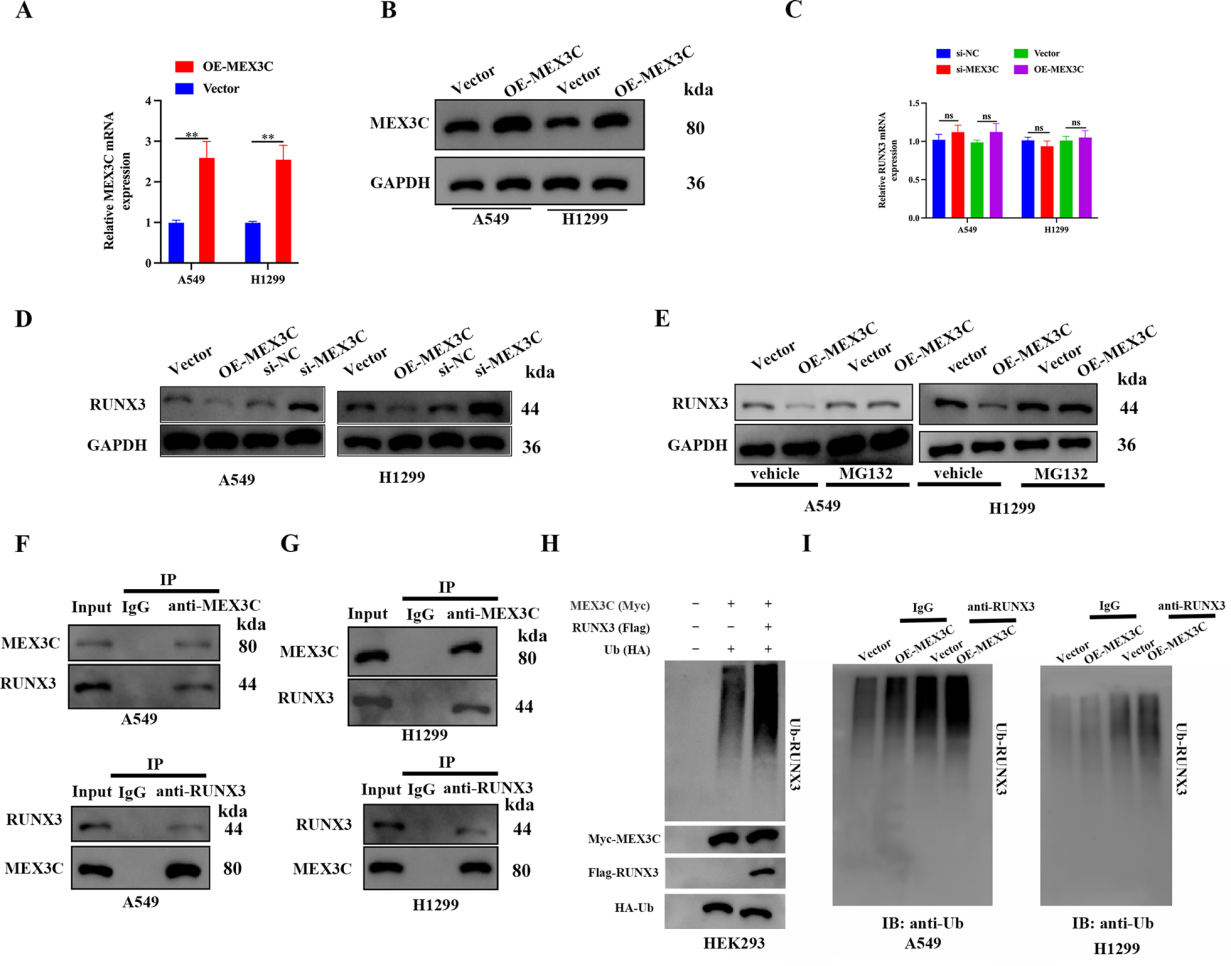
MEX3C mediated RUNX3 ubiquitination and degradation. **A**, **B** Transduction of A549 and H1299 cells with overexpressed MEX3C (OE-MEX3C) or si-MEX3C. Overexpression efficiency of MEX3C in A549 and H1299 cells using qRT-PCR (**A**) and western blotting (**B**). **C**, **D** By using qRT-PCR and western blotting, the mRNA (**C**) and protein (**D**) levels of RUNX3 were determined in MEX3C knockdown or overexpression A549 and H1299 cells. **E** A549 and H1299 cells were transduced with OE-MEX3C and subsequently grown with MG132 at a concentration of 10 μmol/L for 24 h. The presence of RUNX3 protein was determined using western blotting. **F**, **G** The interaction between MEX3C and RUNX3 was detected by Co-IP assay. **H** In vivo ubiquitination assay of HEK293 cells transfected with plasmids expressing Flag-tagged RUNX3, Myc-tagged MEX3C, and HA-tagged ubiquitin. **I** A549 and H1299 cells were transduced with OE-MEX3C. The ubiquitination level of RUNX3 was determined by ubiquitination assays. Bars represent mean ± SD from 3 independent experiments. **P < 0.01. ns, no significance

Having discovered a negative correlation between MEX3C and RUNX3 we next determined whether MEX3C may modify RUNX3 post-translationally. Firstly, qRT-PCR and WB results confirmed that MEX3C overexpression was successful in A549 and H1299 cells (Fig. 4A, B). qRT-PCR addressed that MEX3C knockdown or overexpression had no effect on *RUNX3* mRNA expression (Fig. 4C), but significantly reduced its protein expression, indicating that MEX3C positively regulates RUNX3 at post-transcriptional level (Fig. 4D). In addition, studies have shown that RUNX3 undergoes degradation by ubiquitin-protease system [20]. The inhibitory effect of MEX3C on RUNX3 protein level could be reversed by the proteasome inhibitor MG132 treatment (Fig. 4E), suggesting that MEX3C may be involved in the ubiquitination of RUNX3. Furthermore, Co-IP assay was applied to assess the interaction between MEX3C and RUNX3, and we discovered that MEX3C antibody enriched RUNX3 protein, and RUNX3 antibody can enrich MEX3C protein. Co-IP results showed the interaction between MEX3C and RUNX3 in A549 and H1299 cells (Fig. 4F, G). To determine whether MEX3C can mediate protein ubiquitination in vivo, vectors expressing Myc-tagged MEX3C, HA-tagged ubiquitin, and Flag-tagged RUNX3 were transfected into HEK293 cells. Cell extracts were subjected to immunoblotting with antibodies against Myc, HA, and Flag. MEX3C and ubiquitin were expressed in the transfected cells, resulting in a marked increase in RUNX3 protein ubiquitination (Fig. 4H). A ubiquitination assay conducted with the A549 and H1299 cell lines indicated that the ubiquitination of RUNX3 was greater when MEX3C was overexpressed (Fig. 4I). This evidence supports the proposal that MEX3C promotes tumorigenesis in LUAD by the ubiquitylation and subsequent degradation of RUNX3.

### Inhibiting the expression of RUNX3 could reverse the effects of MEX3C suppression in LUAD cells

**Fig. 5:**
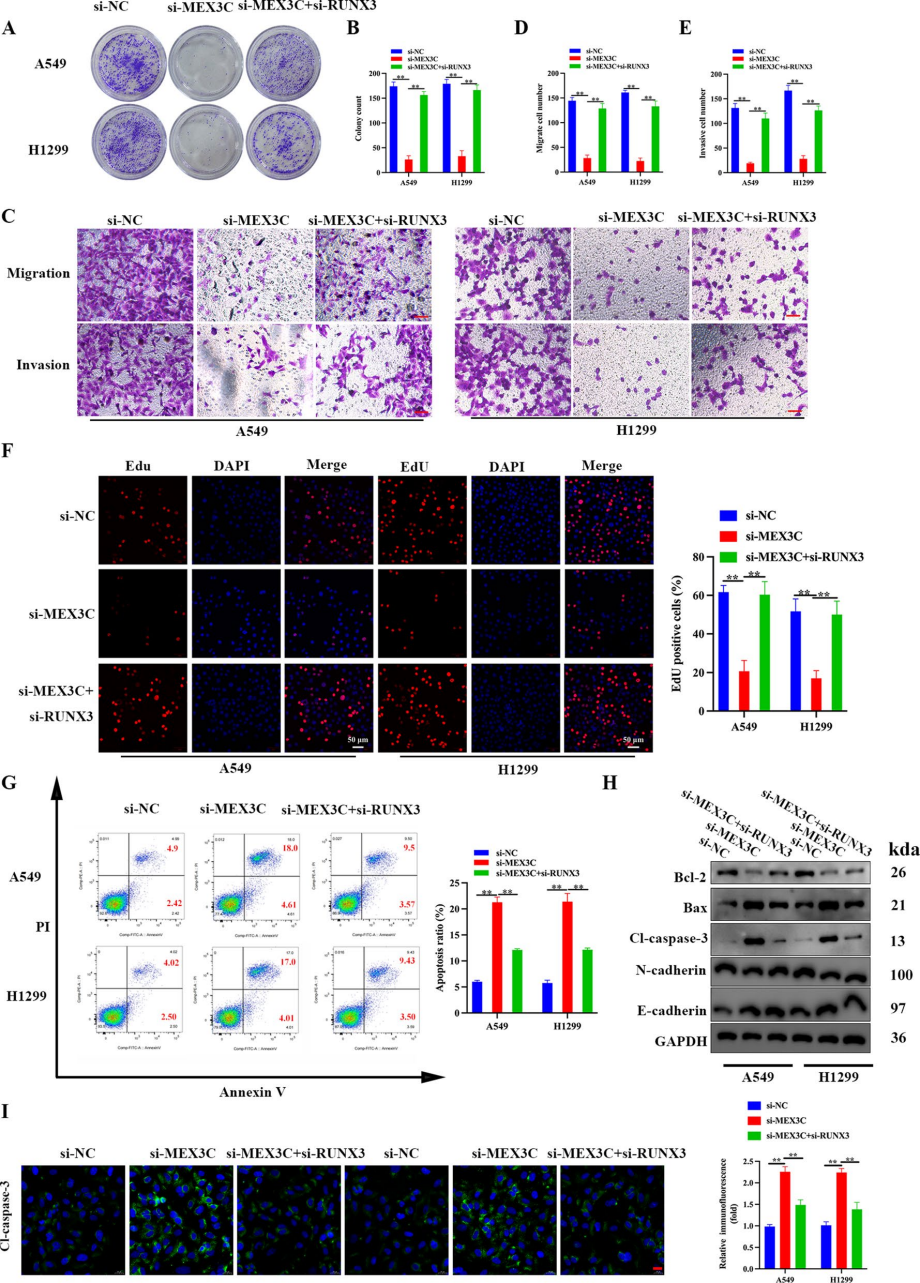
The effects of MEX3C suppression on LUAD cells could be reversed by inhibiting the expression of RUNX3. **A, B** Colony formation was evaluated for A549 and H1299 cells treated with si-MEX3C and/or si-RUNX3. **C–E** Representative images showing the results of Transwell cell migration and invasion assays of A549 and H1299 cells treated with si-MEX3C and/or si-RUNX3, scale bar, 50 μm. **F** Detection of proliferation in A549 and H1299 cells using an EdU assay, scale bar, 50 μm. **G** Flow cytometry assay to detect the cell apoptosis of A549 and H1299 cells treated with si-MEX3C and/or si-RUNX3. **H** Apoptosis and EMT-related protein expression were detected by western blot analysis. **I** Expression of Cleaved-Caspase-3 in A549/H1299 cells as analyzed by immunofluorescence assay, scale bar, 20 μm. Bars represent mean ± SD from 3 independent experiments. **P < 0.01

To verify that the translational modification of RUNX3 by MEX3C was responsible for the oncogenic characteristics of the LUAD cell lines, we silenced the expression of RUNX3 at the same time as suppressing the expression of MEX3C. The suppression of both RUNX3 and MEX3C resulted in a similar level of colony formation to the control LUAD cells (Fig. 5A, B). Similarly, migration and invasion were reduced by the inhibition of MEX3C but inhibiting the expression of both MEX3C and RUNX3 increased levels to that of the control (Fig. 5C–E). In addition, the suppression of both genes increased cell proliferation and apoptosis (Fig. 5F, G) and the expression of apoptotic and EMT-related proteins was similar to those in the control LUAD cells (Fig. 5H, I). This indicates that not only does MEX3C interact with RUNX3 in LUAD cells but other factors controlled by RUNX3 could be contributing to tumor progression.

### RUNX3 transcriptionally downregulates Suv39H1 expression

**Fig. 6:**
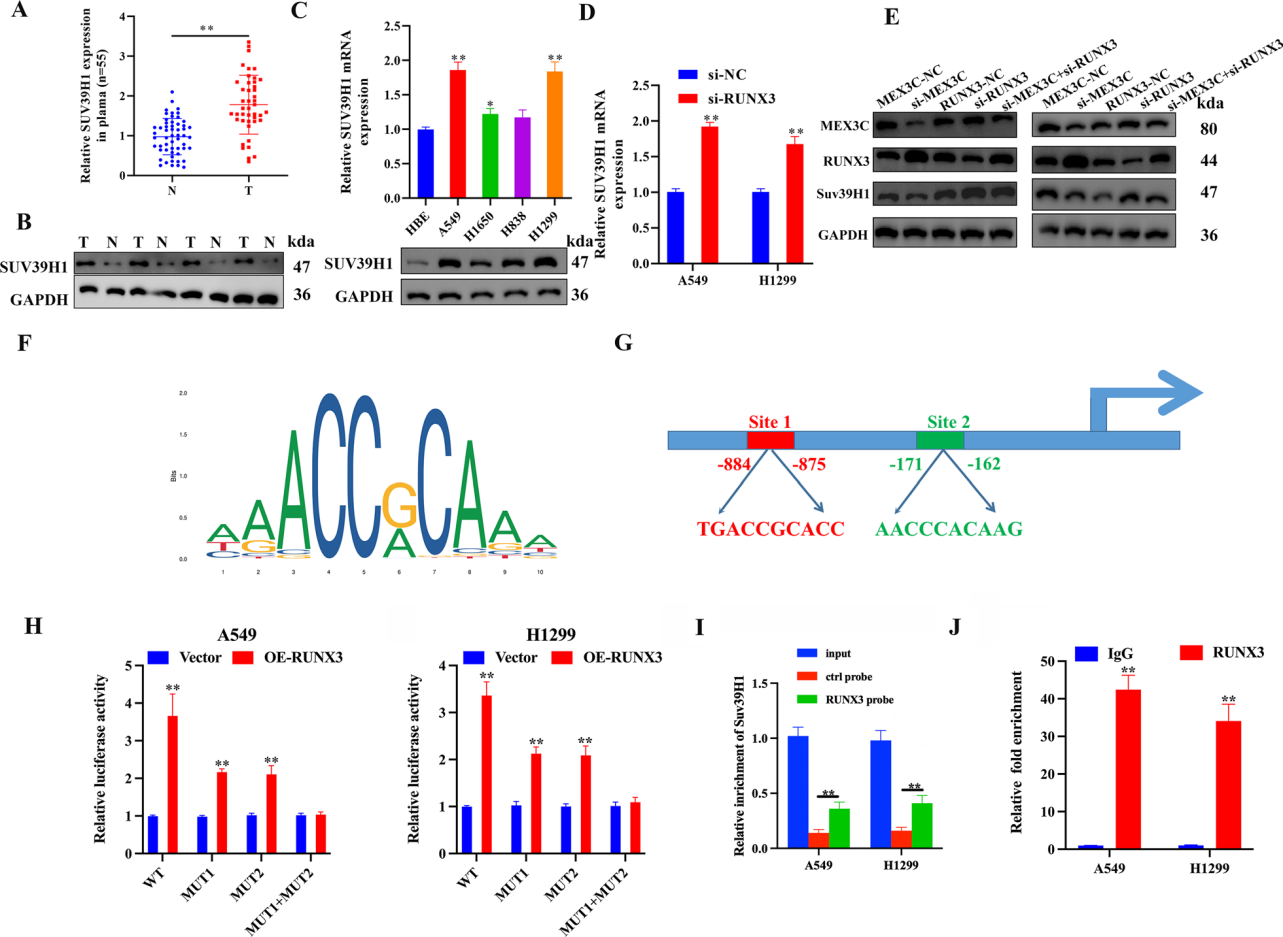
RUNX3 transcriptionally downregulates Suv39H1 expression. **A** *Suv39H1* mRNA levels in 55 LUAD tissues and paired noncancerous lung tissues were detected by qRT-PCR. **B** Protein expression of Suv39H1 in 4 pairs of clinical LUAD specimens. N and T mean adjacent normal tissue and paired lung cancer tissue, respectively. **C** qRT-PCR and western blot analysis of Suv39H1 expression in HBE and LUAD cell lines. **D** qRT-PCR analysis of Suv39H1 expression in A549 and H1299 cells after MEX3C knockdown. **E** Western blot analysis of MEX3C, RUNX3, and Suv39H1 expression in A549 and H1299 cells transfected with si-MEX3C and/or si-RUNX3. **F**, **G** The binding motif of RUNX3 was obtained from JASPAR dataset. Two binding sites for RUNX3 in the Suv39H1 promoter were also predicted by using JASPAR. **H** Assessment of the Suv39H1 promoter RUNX3 binding site via dual luciferase reporter assay. **I** RNA pull-down assay was performed to determine whether RUNX3 targets Suv39H1. **J** ChIP assessment of RUNX3 binding to the Suv39H1 promoter. Immunoprecipitation from A549 and H1299 cells using RUNX3 antibody or mouse immunoglobulin G (IgG). Bars represent mean ± SD from 3 independent experiments. *P < 0.05, **P < 0.01

To understand why the suppression of RUNX3 should promote the progression of LUAD we assessed the expression of its potential target Suv39H1 in patients with LUAD. Suv39H1 is an H3K9 methyltransferase associated with the formation of heterochromatin and known to be expressed at higher levels in NSCLC [29, 30]. *Suv39H1* mRNA expression was lower in normal adjacent tissues than in LUAD tumor tissues (Fig. 6A, B). These results were confirmed in LUAD cell lines with the highest levels of Suv39H1 expression found in A549 and H1299 cells (Fig. 6C). In LUAD cell lines, the mRNA level of *Suv39H1* rose when the expression of RUNX3 was inhibited (Fig. 6D). After interfering with MEX3C in A549 and H1299 cells, the expression of Suv39H1 protein was down-regulated, and the trend of Suv39H1 protein expression was up-regulated after interfering with RUNX3. However, when both MEX3C and RUNX3 were silenced, Suv39H1 protein expression showed very little difference compared to normal controls. This indicates that there is a sequential regulatory link, as the effect was nullified (Fig. 6E). The database USCS (http://genome.ucsc.edu/) was used to obtain the promoter sequence of Suv39H1 and the database JASPAR (https://jaspar.genereg.net/) was used to analyze the potential transcription factors that can bind to the promoter of Suv39H1. We acquired the RUNX3 binding motif and identified two putative RUNX3 binding sites in the *Suv39H1* promoter (Fig. 6F, G). Luciferase reporter assay data showed that upregulating RUNX3 increased the luciferase activity of the wild-type Suv39H1 promoter, whereas mutation of either site 1 or site 2 alone partially attenuated this effect and mutation of both sites totally nullified it (Fig. 6H, I). These results confirmed that RUNX3 interacted with the Suv39H1 promoter at two distinct locations. Meanwhile, RNA pull-down assay using a RUNX3 antibody-conjugated probe showed enrichment of Suv39H1 protein, indicating the interaction between RUNX3 protein and Suv39H1 mRNA. Meanwhile, RNA pull-down assay using a biotin labeled RUNX3 probe could enrich the expression of Suv39H1 protein, indicating the interaction between RUNX3 and Suv39H1 (Fig. 6J). Following this, ChIP assay verified that the RUNX3 antibody had indeed pulled down the Suv39H1 promoter (Fig. 6K). These results suggest that RUNX3 inhibits the transcriptionally-regulated expression of Suv39H1 in LUAD that contribute to tumor development.

### MEX3C promotes tumor growth and accelerates LUAD metastasis in vivo

**Fig. 7:**
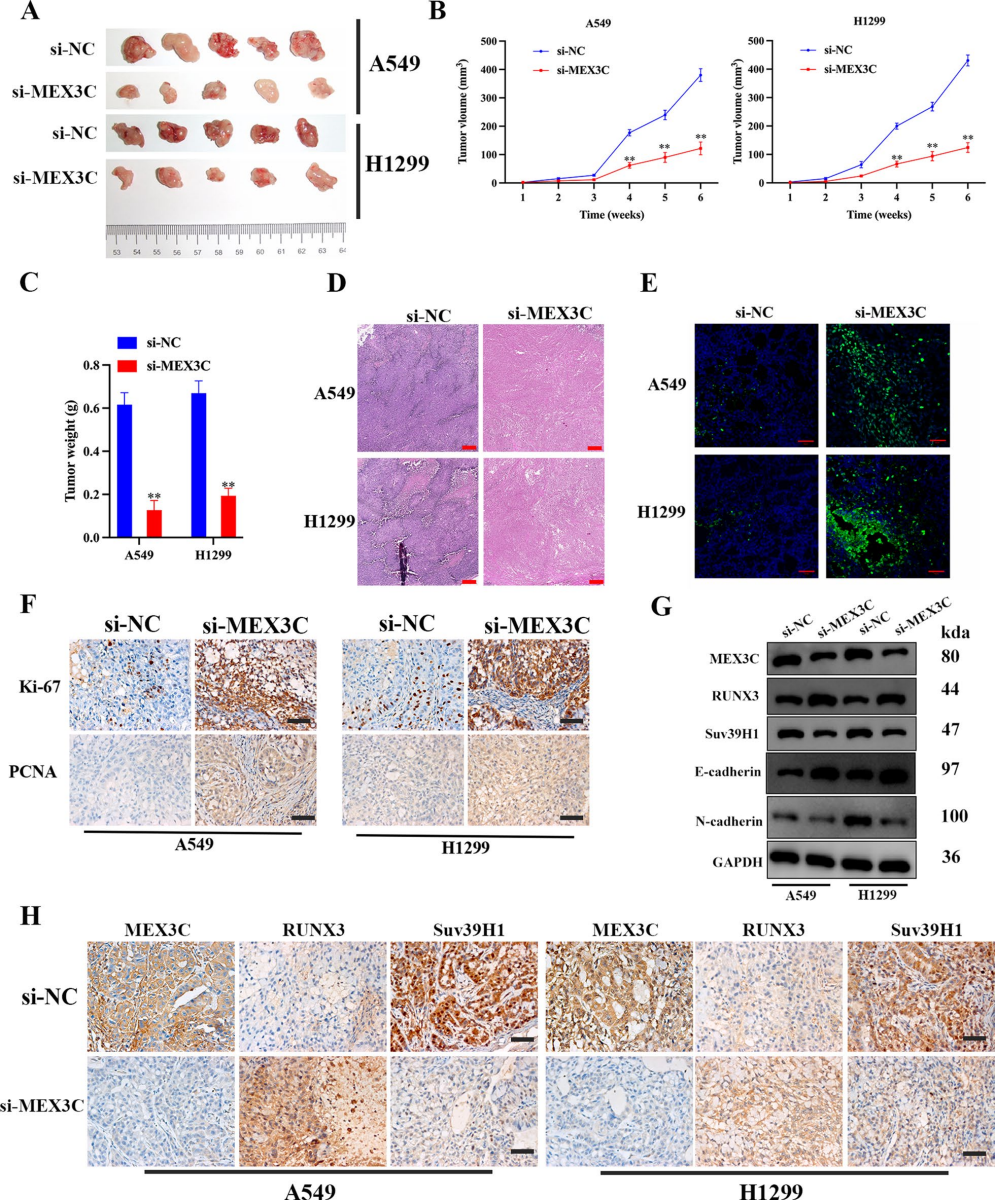
MEX3C promotes tumor growth in vivo. **A** Animal vivo images of MEX3C silencing A549 and H1299 xenograft nude mice (GFP fluorescent images) show that MEX3C silencing reduces the growth of xenografts (n = 5). **A** Images of A549 and H1299 xenograft tumors. **B**, **C** The histograms of tumor volumes and weight revealed that A549 and H1299 cells with MEX3C inhibited generated smaller xenografts when compared to the control cells. **D** The morphology of tumor was determined by H&E staining (scale bar, 200 μm) (**E**). Apoptosis ratio was determined by TUNEL assay (scale bar, 200 μm). **F** Immunohistochemistry was used to determine and compare the expression levels of Ki-67 and PCNA between the si-NC and si-MEX3C groups. Scale bar, 100 µm. **G**, **H** Western blot analysis and Immunohistochemistry were used to determine the protein levels of MEX3C, RUNX3 and Suv39H1 as well as EMT-related proteins in tumors of mice models of si-NC and si-MEX3C groups. Data were expressed as mean ± SD, n = 5, **P < 0.01

To determine the influence of MEX3C on the promotion of tumorigenesis in vivo, we created an animal model using si-MEX3C transfected A549 or H1299 cells. Tumor volume and weight were significantly reduced by the suppression of MEX3C in both LUAD cell lines (Fig. 7A–C). H&E staining revealed that si-MEX3C treatment showed significant morphological changes compared to the si-NC group (Fig. 7D). In addition, TUNEL staining showed that the number of green-stained positive cells was significantly decreased after treatment with si-MEX3C compared with the si-NC group, indicating that MEX3C significantly promoted apoptosis of tumor cells (Fig. 7E). Ki-67 is expressed in all stages of cell division but is absent in non-dividing cells whereas PCNA is associated with DNA synthesis, both are used widely as cell proliferation markers [31]. We measured levels of the Ki-67 and proliferating cell nuclear antigen (PCNA) in tumor tissue by immunohistochemistry assay (Fig. 7F). Levels of Ki-67 and PCNA were increased in tumor tissue when MEX3C was silenced. In addition, western blot analysis and/or immunohistochemistry assay found that RUNX3 and E-cadherin was upregulated when MEX3C was inhibited, and expression of Suv39H1 and N-cadherin was decreased when MEX3C was inhibited indicated that EMT was suppressed (Fig. 7G, H).

### MEX3C accelerates LUAD metastasis in vivo

**Fig. 8:**
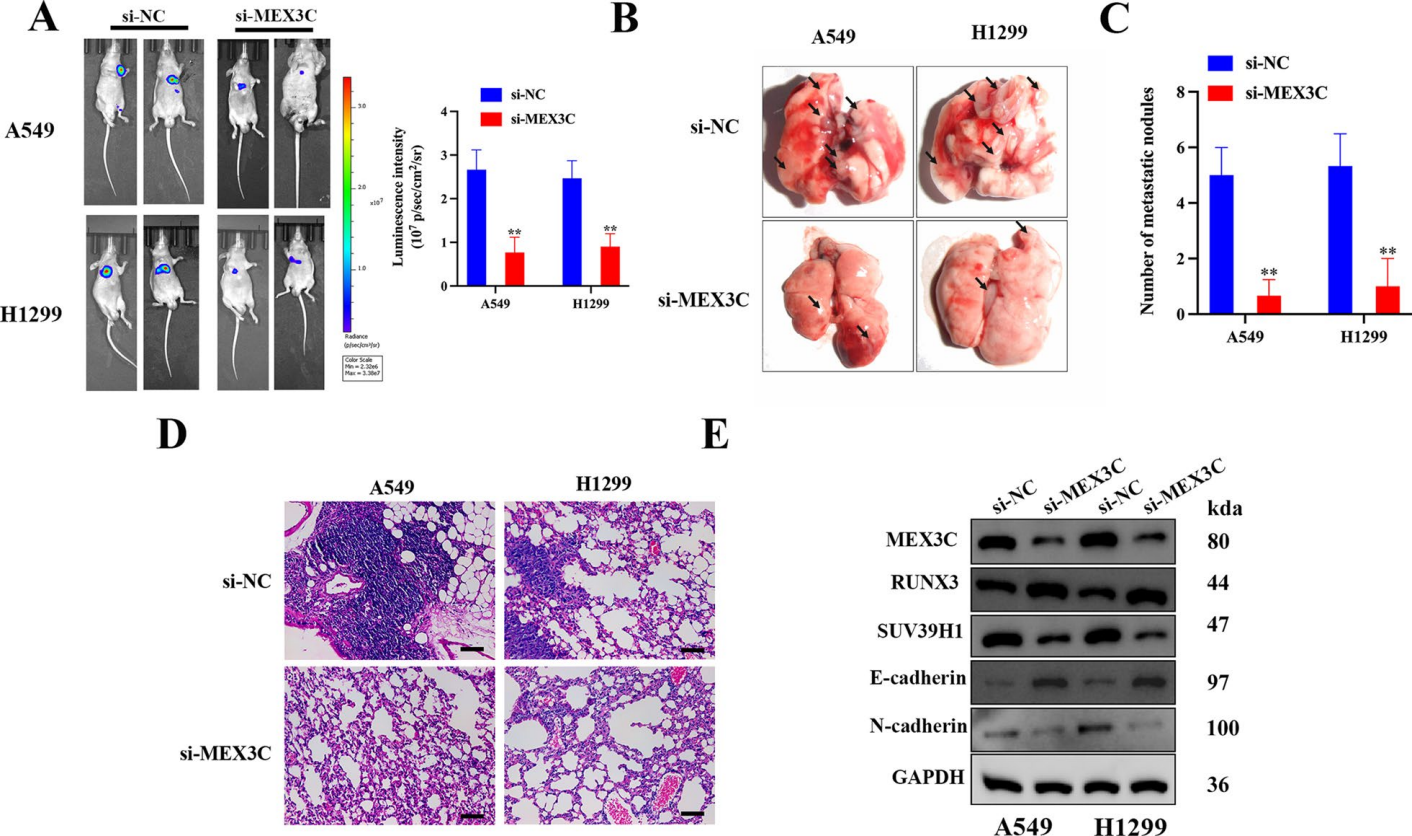
MEX3C accelerates lung cancer metastasis in vivo. **A** Nude mice were injected subcutaneously with A549 and H1299 cells expressing either si-NC or si-MEX3C. The tumor metastasis was detected by using IVIS^®^ bioluminescence imaging system 8 weeks after injection. Quantitative data of tumor metastases detected by IVIS system. **B**, **C** The lungs were removed and imaged 8 weeks after injection via the tail vein. The number of metastatic nodules in the lungs was counted. **D** The H&E staining of lung tissues from different group of mice, scale bar, 50 μm. **E** Western blot analysis was performed to detect the protein expression of MEX3C, RUNX3, and Suv39H1, E-cadherin and N-cadherin. Data were expressed as mean ± SD, n = 5, **P < 0.01

Luciferase activity confirmed that the expression of MEX3C was reduced in mice injected with LUAD cells transfected with si-MEX3C (Fig. 8A). Figure 8B indicates that the metastatic spread of tumor nodules in the lungs of mice is more severe in those expressing MEX3C. Inhibiting the expression of MEX3C results in the reduction of metastatic nodules (Fig. 8C). In the tumor tissue derived from A549 and H2199 NSCLC cells, protein levels of RUNX3 were increased when MEX3C was inhibited but Suv39H1 protein levels were lower, indicating that RUNX3 could negatively control Suv39H1 (Fig. 8D). Western blotting results of the tumor tissue also confirmed that proliferation and EMT were inhibited by the suppression of MEX3C. The results above indicated that MEX3C could accelerates lung cancer metastasis in vivo.

## Discussion

As one of the most prevalent malignancies, lung cancer is a deadly disease with a 5-year survival rate of approximately 21% [2]. NSCLC accounts for the majority of lung cancer but it has a low survival rate because it is diagnosed predominantly at a later stage when metastasis can occur [32, 33]. LUAD, accounting for approximately 40% of lung malignancies, is the most known subtype of lung cancer. Moreover, the treatment of LUAD is complicated by the heterogeneity of tumors and a diverse number of genetic profiles [34, 35]. Therefore, therapeutic approaches involving posttranslational modifications such as ubiquitylation may result in targets with greater specificity in NSCLC [36].

MEX3 family are implicated in numerous biological processes that contribute to the occurrence and progression of cancer [13]. Numerous investigations demonstrate the oncogenic roles of MEX3 family proteins in various cancers [11, 12, 37, 38]. MEX3C, which is occasionally referred to RKHD2, is a member of the Mex-3 protein family. MEX3A-D, are the four different protein members that make up the Mex-3 protein family [8]. According to the findings of research, MEX3A has the potential to act as both a predictive biomarker and a target for metastatic therapy in LUAD [15]. Despite the fact that it is widely expressed in a variety of tissues, however, very little is known about the role that another essential member plays in cancer: MEX3C. Other study has suggested that MEX3C is involved in a number of different biological processes, including immunological responses [39], the transfer of RNA molecules [40], the suppression of translation [41], and energy balance [42]. On the other hand, there is not a lot of information available about MEX3C and its connection to human LUAD at this time. In this study, we investigated the consequences of MEX3C ubiquitylation on RUNX3 LUAD. We found that MEX3C is upregulated in LUAD tissue and cells, especially in A549 and H1299 cell lines. We also found that knockdown of MEX3C could inhibit LUAD proliferation, migration and invasion and promote apoptosis in vitro and in vivo. Therefore, MEX3C is a new oncogene in LUAD, and may be a new drug candidate for use in LUAD therapeutics.

The RUNX3 gene is situated on the chromosomal region 1p.13-p36.11, which is recognized as a deletion hotspot in several types of malignancies originating from epithelial, hematological, and neural tissues [43]. RUNX3 inactivation, as found in this study, has been associated with the progression of several cancers including NSCLC [44–48]. RUNX3 suppression is thought to lead to the activation of the S phase in the cell cycle. Therefore, the combined loss of RUNX3 and p53 would result in greater tumor progression and would explain the elevated levels of tumor characteristics that we discovered in H1299 cells [47]. In several cancers, such as breast, colorectal and gastric cancers, the mis-localization of RUNX3 to the cytoplasm is thought to result in tumor progression; however, in other cancers including NSCLC, it was found that the loss of RUNX3 expression through posttranslational modification leads to tumor progression [44]. RUNX3 is known to be a downstream effector of the transforming growth factor-β (TGF-β) signaling pathway, which is involved in apoptosis, angiogenesis, EMT, cell migration, and invasion [43].

In this study, we found that the inactivation of RUNX3 occurs through ubiquitylation by MEX3C. This in turn causes a higher expression of Suv39H1, one of the RUNX3 target genes. The ubiquitination of proteins is a multistep process that is regulated by proteins that belong to enzyme families E1, E2, and E3 [49]. Erroneous ubiquitylation often leads to the progression of proliferation and metastasis in NSCLC [36]. Ubiquitylation by upregulated MEX3C is involved in the progression of hepatocellular carcinoma [50]. According to the findings of our studies, MEX3C promotes tumorigenesis in LUAD by the ubiquitylation and subsequent degradation of RUNX3. Notably, a similar mechanism of tumorigenesis regulation via ubiquitylation has been reported for MEX3A in glioblastoma [51]. This recent study demonstrated that MEX3A induces the ubiquitylation and proteasomal degradation of the tumor suppressor RIG-I in glioblastoma, resulting in increased cell growth. Given the parallels with our findings, therapeutically targeting ubiquitylation enzymes like MEX3C and MEX3A may hold promise as an innovative strategy to suppress tumorigenesis in cancers where they play an oncogenic role.

Our research indicates that RUNX3 is a MEX3C target and that the loss of RUNX3 expression leads to a higher expression of Suv39H1. Suv39H1 is a protein lysine methyltransferase that is implicated in several cancers, including NSCLC [30], and is also involved in EMT [52]. The inhibition of Suv39H1 can prevent EMT in breast cancer through the depletion of H3K9me3 in the promoter region of E-cadherin [53]. A similar mechanism could occur in our study when Suv39H1 is suppressed by RUNX3.

Our study also has some limitations. To minimize the use of animals, a low sample size was used to determine the level of significance obtained with in vivo studies. However, these experiments were conducted primarily to establish whether the proliferation of tumors and metastasis could occur in vivo.

## Conclusions

In the present study, we have demonstrated that MEX3C is upregulated in LUAD tumor tissue whereas RUNX3 is downregulated. Our results indicate that MEX3C may be involved in the ubiquitylation of RUNX3 to promote the tumorigenesis of LUAD cells. RUNX3 may regulate the proliferation and EMT status of cells through interacting with downstream targets that include Suv39H1. Our results suggest that inhibiting or suppressing the activity of MEX3C could be useful in the management of LUAD.

## Data Availability

All data generated or analyzed during this study are included in this published article.

## Abbreviations

LUAD: Lung adenocarcinoma
NSCLC: Non-small cell lung cancer
LUSC: Lung squamous cell carcinoma
MEX3: Muscle Excess 3
OC: Ovarian cancer
TCGA: The Cancer Genome Atlas
GTEx: Genotype-Tissue Expression
HBE: Human bronchial epithelial
P/S: Penicillin/streptomycin
FBS: Fetal bovine serum
CCK-8: Cell Counting Kit-8
FITC: Fluorescein isothiocyanate
Pi: Propidium iodide
TUNEL: Terminal deoxynucleotidyl transferase dUTP Nick-End Labeling
EdU: 5-Ethynyl-2′-deoxyuridine
H&E: Hematoxylin and eosin
qRT-PCR: Reverse transcription-quantitative polymerase chain reaction
CO-IP: Co-immunoprecipitation
CHIP: Chromatin immunoprecipitation IP
TBST: Tris-buffered saline with 0.1% Tween
CEA: Carcinoembryonic antigen
SCCAg: Squamous cell carcinoma antigen
EMT: Epithelial–mesenchymal transition
